# The *ACTN-3* c.1729C>T (rs1815739) Polymorphism Is Associated with Match-Play Maximal Running Speed in Elite Football Players: A Preliminary Report

**DOI:** 10.3390/sports13090331

**Published:** 2025-09-16

**Authors:** Myosotis Massidda, Laura Flore, Giovanna Maria Ghiani, Kinga Losinska, Mauro Baldus, Jacopo Secci, Giuseppe Allegra, Marco Scorcu, Naoki Kikuchi, Pawel Cieszczyk, Carla Maria Calò, Filippo Tocco

**Affiliations:** 1 Department of Medical Science and Public Health, University of Cagliari, 09042 Monserrato, Italy; giovannam.ghiani@unica.it (G.M.G.); filippo.tocco@unica.it (F.T.); 2 Faculty of Physical Education, Gdansk University of Physical Education and Sport, 80-336 Gdansk, Poland; kinga.losinska@awf.gda.pl (K.L.); pawel.cieszczyk@awf.gda.pl (P.C.); 3 Department of Life and Environmental Sciences, University of Cagliari, 09042 Cagliari, Italy; laura.flore@unica.it (L.F.); cmcalo@unica.it (C.M.C.); 4 Cagliari Calcio SpA, 09122 Cagliari, Italy; maurobaldus@gmail.com (M.B.); jacoposecci@me.com (J.S.); giuseppe.allegra92@tiscali.it (G.A.); mscorcu@tiscali.it (M.S.); 5 Italian Sport Medicine Federation (FMSI), CR Sardegna, 00196 Rome, Italy; 6 Graduate School of Health and Sport Science, Nippon Sport Science University, Tokyo 158-8508, Japan; n.kikuchi@nittai.ac.jp

**Keywords:** alpha-actnin 3, gene, velocity, soccer, sprint performance

## Abstract

The TT genotype of the *ACTN-3* polymorphism (rs1815739) has been previously associated with lower sprinting and jumping performance, higher frequency and severity of muscle injuries and eccentric muscle damage in professional football players. This study examined the influence of rs1815739 polymorphism on maximal running speed (MRS) during official matches in elite football players. MRS was collected, using a Global Position System (GPS) at high sampling frequencies (50 Hz), from 45 footballers of the same team during 26 official matches (707 match observations). A buccal swab was used to extract genomic DNA, and an RFLP PCR technique was used to determine the *ACTN-3* genotype. The main finding of the present study was that CC players showed significantly higher MRS than TT players (CC = 33.1 ± 1.3 km·h^−1^; CT = 32.7 ± 1.6 km·h^−1^; TT = 31.5 ± 1.9 km·h^−1^, *p* = 0.041). Moreover, the players harboring a copy of the C allele showed a trend toward higher MRS than TT genotype (CC + CT = 32.9 ± 1.5 km·h^−1^ vs. TT = 31.5 ± 1.9 km·h^−1^, *p* = 0.06). We found, for the first time, an association between the *ACTN-3* polymorphism and match-play MRS in elite football players. Our results bring new knowledge to the literature regarding the advantage conferred by the C allele (CC and CT genotypes) of the *ACTN-3* polymorphism on sprint performance in football providing perspectives for modulating the speed training program in relation to *ACTN-3* genotypes, enhancing performance avoiding muscle lesions.

## 1. Introduction

Athletic performance is one of the most complex phenotypic variables that is influenced by both hereditary and environmental influences. Genetic variables account for an average of 66% of the variance in athlete status [1], with environmental factors—such as nutrition and training—accounting for the remaining variance [2,3]. According to a recent study by Semenova et al. [4], a total of 251 DNA polymorphisms have been linked to athlete status and, among them, the *ACTN-3* c.1729C>T (rs1815739) polymorphism is one of the most promising genetic markers.

The *ACTN-3* c.1729C>T (rs1815739) polymorphism is localized in Chromosome 11 and it codifies for the alpha-actinin-3, a binding protein localized in the Z-disc of the type II muscle fibers, where it facilitates the myofibrillar actin filaments’ anchoring [5]. The T homozygous genotype (*ACTN-3* 577TT) is caused by a C to T nucleotide substitution in exon 16 of the *ACTN-3* gene, which leads to a change in the codon (from CGA to TGA), resulting in a premature stop codon that causes the absence of the alpha-actinin 3 protein in muscle fibers [6,7]. Among individuals homozygous for the 577T allele (TT genotype), the lack of alpha-actinin 3 does not indicate any illness or clinical condition. On the other hand, homozygous individuals with the 577C allele (CC genotype) or heterozygous individuals with the CT genotype express α-actinin-3 in a dose-dependent manner (CC > CT) [8].

Higher strength and sprint/power performance have been connected to the wild-type alleles, C or R, while improved muscle endurance performance has been linked to the mutant alleles, T or X [9,10].

According to several studies, power/speed athletes are more likely than endurance athletes or the general population to have higher CC genotype and C allele frequencies [11,12,13,14]. Therefore, it appears that the C allele and CC genotype confer an advantage in producing strong, forceful muscular contractions, which are necessary for sprinting performance.

Researchers have focused a lot of attention on the relationship between *ACTN-3* polymorphism and football, discovering a link between this genetic marker with muscle damage and injuries [15,16,17], football performance [18,19,20,21] and football player status [22].

In more detail, the CC genotype was found to be significantly associated with a lower incidence and severity of muscle injuries [16] with recovery times [17], and with increased susceptibility to eccentric muscle damage [15] in elite football players.

Moreover, a recent systematic review and meta-analysis by McAuley et al. [22] found significant correlations between professional footballer status and the presence of the *ACTN-3* C allele (OR = 1.35, 95% CI: 1.18–1.53), while the strongest associations were found for the *ACTN-3* CC genotype (OR = 1.48, 95% CI: 1.23–1.77). The CC genotype has also been associated with sprinting speed and jumping performance in Brazilian [19,20], Italian [18] and Turkish [21] professional football players, while the TT genotype seems to negatively affect football career progression [23].

These findings could be explained by the link between the *ACTN-3* TT genotype and the higher frequency and severity of non-contact muscle injuries and muscle damage and the association between the *ACTN-3* CC genotype and power-oriented phenotypes.

High-intensity running is one of the essential components of modern football and can set top players apart from those at a lower level. [24]. Players need to build a strong capacity to undertake intense activities at maximal running speed because most goals are scored following a fast run or a sprint by both the supporting player and the goal scorer [25]. The requirement for quick and skilled players is highlighted by the 15% rise in game speed observed in FIFA World Cup Finals analyses from 1966 to 2010 [26]. Consequently, in contemporary football, managers are increasingly looking for players who are fast, skilled, and physically fit enough to undertake high-intensity exercises repeatedly, as well as who can handle a heavy weekly training and game load [27].

One study recently investigated the influence of the *ACTN-3* polymorphism on match running performance in professional football players using the multicamera Mediacoach^®^ system (Spain) [28]. The authors found that TT players had lower match performance values than CC players in the total running distance (*p* = 0.046), the distance at 21.0–23.9 km/h (*p* = 0.042), and the number of sprints (*p* = 0.042).

The current pilot study sets out to investigate, for the first time, the influence of the ACTN-3 c.1729C>T (rs1815739) polymorphism on elite professional football players’ maximal running speed performance (MRS) during official games utilizing the Global Positioning System (GPS). We hypothesized that football players harboring the C allele achieve a higher MRS during matches than those footballers with the TT genotype. The C allele is hypothesized to lead to higher MRS due to the role of alpha-actinin-3 alpha-ctinin-3 protein that is exclusively expressed in fast-twitch (Type II) muscle fibers and modifies skeletal muscle function [10].

## 2. Materials and Methods

### 2.1. Participants

A total of 45 elite football players from a professional team competing in the first division of football in Italy (i.e., Serie A and Primavera) participated in this study. All the football players were of Caucasian descent for at least 3 generations. In the sample, there were 11 forwards, 19 midfielders and 15 defenders. Written informed consent was obtained from all the players. The University of Cagliari’s local ethical committee approved the study’s protocol on 30 January 2017, in compliance with the International Federation of Sports Medicine’s consensus statement regarding genetic information and the 1974 Declaration of Helsinki for Human Research, which was last updated in 2000 [29].

### 2.2. Genetic Testing

Genetic data were collected between July and August 2023. Genomic DNA was obtained by buccal cells with a cotton swab, and it was extracted in October 2023, according to the manufacturer’s directions provided with a commercially available kit (Qiagen S.r.l., Milan, Italy) [30]. Exon16 of the *ACTN-3* polymorphism was amplified through polymerase chain reaction (PCR) using the following primers:

forward 5′-CTGTTGCCTGTGGTAAGTGGG-3′
reverse 5′-TGGTCACAGTATGCAGGAGGG-3′.

The DdeI enzyme was used to digest PCR products. The presence (577T) or the absence (577C) of a DdeI restriction site in exon 16 identified the 577C and 577T alleles (CGA and TGA codons, respectively). While allele 577C displays three fragments of 108, 97, and 86 bp, allele 577T displays two fragments with 205 and 85 bp. After separating the resulting fragments using 10% polyacrylamide gel electrophoresis, Eurosafe solution (produced by Euroclone S.p.A.) was used to stain them [30].

### 2.3. Maximal Running Speed (MRS) During Official Matches

MRS (km·h^−1^) performance was obtained from all players in the matches played in the 2023–2024 season (from 19 August 2023 to 26 May 2024) using the Global Position System (GPS) (KSport^®^ World Srl, Fano, Italy) with a sampling frequency of 50 Hz and previously validated [31]. Numerous technical and educational studies of GPS have already been published, describing how this technology allows for tracking three-dimensional movement of individuals [32,33,34]. The expanded use of this technology in a range of contexts, including football, has been made possible by the recent advent of portable GPS devices, which offers another way to characterize and comprehend the spatial context of football performance. With enough sensitivity to detect speed variations and analyze the physical demands [35] and movement patterns [36] of professional football, GPS devices are a proven and efficient tool that can be used to measure physical demands during a football match [37]. The GPS was located in a vest that was properly fitted to the wearer’s body at the upper back. At the conclusion of each match, GPS-measured Maximal Running Speed (Vmax) data was downloaded into an Excel spreadsheet. Players having fewer than 90 min of match exposure over the course of the season were excluded from the study since their running data was probably not indicative of their match performance. The match exposure was calculated by adding up each player’s total minutes on the field (first, second, and extra time) as derived from GPS output. The analyses contained only the data of players who played for more than 90 min during the season. A subsample of 37 football players was obtained after players who had played in fewer than 90 min of games during the entire season were removed.

### 2.4. Statistical Analyses

Fisher’s method (χ2) was used to determine the Hardy–Weinberg equilibrium (HWE) for the *ACTN-3* polymorphism. Additionally, a χ2 test was carried out to confirm if the genotype frequency in our football player group differed from that of ethnically matched controls in the 1000 Genomes database (1000 Genome Database). For every genotype, descriptive statistics were computed as mean ± standard deviation for continuous variables and frequencies for categorical variables. χ2 tests were also used to identify genetic differences for categorical variables (such as field position). The Kolmogorov–Smirnov test was used to first verify normality for continuous variables (such as Vmax or anthropometric variables), and a one-way analysis of variance (ANOVA) was used to compute genotype differences in order to identify the primary effect of the *ACTN-3* genotype. LSD post hoc tests were used to find pairwise differences (CC vs. CT; CC vs. TT; CT vs. TT) where there was a significant F test indicating a main effect of the genotype. Furthermore, unpaired Student’s t-tests were used to determine the impact of a dominant model for the *ACTN-3* genotype (CC vs. CT + TT) and a recessive model (CC + CT vs. TT) on the MRS performance measure. For all statistical analyses, the significance level was set at *p* < 0.050. All statistical analyses were performed with statistical software (Genepop v.4.8.2 and STATSoft V7).

## 3. Results

All variables had a normal distribution (*p* > 0.05). With the following genotype distribution—CC, 40.5%; CT, 43.2%; and TT, 16.2%—the *ACTN-3* polymorphism did not deviate from HWE and the genotyping success rate was 100% (Table 1).

Our sample of professional football players had genotype frequencies that were comparable to those of the Caucasian population in the 1000 Genomes database (*p* = 0.484). Age, body height, body mass, and match exposure did not differ between genotypes (Table 1).

**Figure 1:**
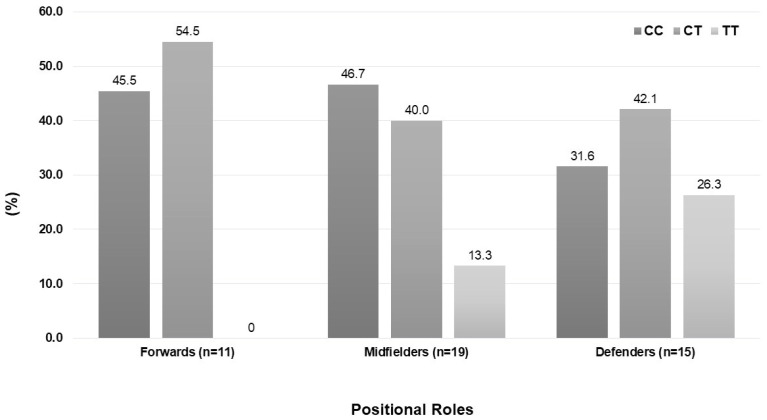
Distribution (%) of *ACTN-3* genotypes in 45 professional football players according to their positional roles.

Figure 1 shows the *ACTN-3* genotype distribution according to the field position in the sample of 45 football players. Although the genotype distribution did not differ significantly among the various field positions (*p* = 0.435), it is noteworthy that no forward football player had the TT genotype.

There was a main effect of the genotype on MRS performed during the official matches in the subsample of 37 football players (Table 2).

**Figure 2:**
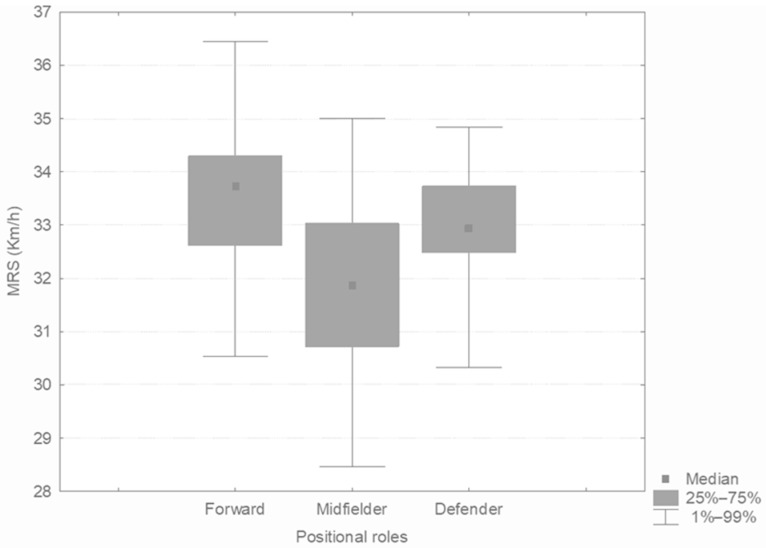
MRS (km·h^−1^) according to positional roles.

TT players had a significantly lower MRS than CC players (CC = 33.1 ± 1.3 km·h^−1^, CT = 32.7 ± 1.6, TT = 31.5 ± 1.9 km·h^−1^, *p* = 0.041; Figure 2). A trend toward higher MRS was also observed in players with a copy of the C allele compared to those with the TT genotype (CC + CT = 32.9 ± 1.5 km·h^−1^ versus TT = 31.5 ± 1.9 km·h^−1^, *p* = 0.06). A players with the CC genotype recorded the higher maximal running speed (36.4 km/h^−1^), while a player with the TT genotype showed the lower maximal running speed (29.8 km/h^−1^).

As a secondary finding, Figure 2 shows the MRS according to positional roles. Midfielders showed a significantly lower MRS than forward players (Forwards, 33.5 ± 1.6 km·h^−1^ vs. Midfielders, 31.9 ± 1.7 km·h^−1^, *p* = 0.02).

## 4. Discussion

Examining the impact of *ACTN-3* polymorphism on professional football players’ maximum running speed during official games was the primary goal of this study. Although previous studies have shown an association between the *ACTN-3* polymorphism with football player status [38] and some key factors of football performance, such as high intensity running during match play [28] sprinting and jumping [19], none of them have analyzed the maximal running speed during official matches using global position system (GPS) data collection.

Recent studies performed using the GPS technology showed that physical demands are ever increasing in football, particularly the number of high-speed runs and sprints [39]. Additionally, it has been noted that contextual factors like the playing position affect sprint and acceleration-related characteristics (such as maximal speed or accelerations) [40].

The results of this study are in line with those of Del Coso et al. [41], who found that the fastest players were forwards, with a maximum running speed of 33.03 ± 1.35 km/h, followed by defenders (32.72 ± 1.32 km/h; *p* = 0.025) and midfielders (32.08 ± 1.63 km/h; *p* < 0.001).

Regarding the influence of the *ACTN-3* polymorphism on MRS, the major finding of the present work was that football players carriers of CC genotype showed a significantly higher MRS than TT players. Furthermore, compared to players who carried the TT genotype, those who possessed at least one copy of the C gene exhibited a tendency toward higher MRS.

Our results agree with the study conducted by Del Coso et al. [28] that evaluated the influence of *ACTN-3* polymorphism on football running speed performance during matches collected using a multicamera Mediacoach^®^ system (Madrid, Spain) [28]. The authors found that the professional football players with the TT genotype had a lower match running performance, especially in variables associated with high-intensity running and sprinting, than their CC counterparts.

In our previous study [18], we found that the CC genotype was associated with explosive power in Italian top-level football players, such as replicated in a study on Turkish players [21]. The CC genotype was also found to be significantly associated with higher quadriceps and hamstring strength [42], lower muscle injuries [16], football player status [22], better jumping and sprinting performance [18,38], career progression [23], and, more recently, with higher post-match creatine kinase (CK) levels and a higher number of sprints during a match [43]. Moreover, the CC genotype was also associated with sprinting speed and explosive power in Brazilian youth and professional players [19,20,44].

More in general, the C allele seems to influence the ability to express strong muscle contractions at high speeds [6,45], giving an advantage to elite athletic performance in power and sprint sports (events involving running, jumping, and throwing) [9]. Several investigations have confirmed the link between sprint performance and the C allele and the CC genotype in populations with a variety of ethnic backgrounds, including Russian [46]; European [38,47], American [48]; Greek [49]; Israeli [11], Japanese [50], Australian and Brazilian athletes [51]. Consequently, it is highly probable that α-actinin-3 deficiency impairs rapid skeletal muscle fiber performance in both elite athletes and the general population [45].

Furthermore, the *ACTN-3* TT genotype causes fast-type muscle fibers to lack α-actinin-3, which would result in a weaker connection between the Z-line and actin filaments. This could cause a structural deficit that makes a sarcomere more vulnerable to damage from high mechanical stress, such as sprint training.

Stolen et al. [52] describe soccer as a physically demanding team sport with an intermittent activity profile that alternates high-intensity activities like sprinting, jumping, tackling, and accelerations with low-intensity phases of active recovery (such as walking, jogging) and passive recovery (such as standing). One of the most important skills for soccer players to compete at the professional level is the capacity to achieve and maintain high-speed running and sprint [53].

Male soccer match play intensity has increased over the past 15 years, primarily due to increased demands for high-speed running (distance covered at speeds between 19.8 km·h^−1^ and 25.1 km·h^−1^) and sprinting (distance > 25.1 km·h^−1^), which now account for roughly 7–11% and 1–3% of the total distance covered during a match, respectively [54,55,56]. Additionally, sprint and high-speed running movements are thought to be significant variables for an optimal football performance [24]. For example, straight sprinting, which can be done by the football player who scores or the one who assists, has been found to be the most common locomotive action that occurs before goal scenarios [25,57]. Therefore, it is essential for professional soccer coaches to strengthen their players’ high-speed running and sprinting abilities.

The small sample size used for the analysis is the main limitation of the current study; therefore, additional research is required to replicate the findings in other football player cohorts. However, the points of strength are represented by the high number of observations made throughout the matches (707 observations), the approach used to gather the highest running speed, and the exceptional homogeneity of the sample under study. Additionally, only elite football players from the same team were included in the analysis of the sample chosen for this study. Because football performance is influenced by the training methodology, the pitch, the athletes’ age, and their skill level, we think it is crucial to reduce as much as possible the number of those confounding variables, even if it means a smaller sample size [58].

## 5. Conclusions

Our preliminary data suggest, for the first time, that the *ACTN-3* polymorphism is associated with maximal running speed during match-play in elite football players. The main findings of the present study were that CC players showed significantly higher maximal running speed achieved during match than TT players. Moreover, the players harboring a copy of the C allele showed a trend toward higher maximal running speed than players carriers of the TT genotype. Our finding adds to the existing literature, bringing new knowledge on the role of *ACTN-3* gene as genetic marker associated with sprint performance in football. Knowing the *ACTN-3* genotype of their football players can help coaches adjust their training program to best fit the player’s genotype, maximizing their speed and avoiding health issues like muscle injuries.

## Figures and Tables

**Table 1:** Main characteristics of 45 professional football players according to their *ACTN-3* genotype.

Variables	CC	CT	TT	*p* Value
Number (observed frequency, %)	18 (40.9)	20 (45.4)	7 (15.9)	0.433
Number (expected frequency, %)	17.3 (38.4)	21.3 (47.3)	6.3(14)	
Age (years)	20.4 ± 5.7	19.3 ± 3.1	22.0 ± 6.4	0.315
Height (cm)	180.7 ± 6.6	177.5 ± 8.5	178.2 ± 11.2	0.717
Weight (kg)	74.1 ± 9.2	72.0 ± 6.7	71.8 ± 12.7	0.531

**Table 2:** Main characteristics of the 37 professional football players according to their *ACTN-3* genotype.

Variables	CC	CT	TT	*p* Value
Number (frequency, %)	15 (40.5)	16 (43.2)	6 (16.2)	0.484
Age (years)	21.1 ± 6.0	19.8 ± 3.3	23.3 ± 5.9	0.345
Height (cm)	181.8 ± 6.0	176.6 ± 8.1	181.0 ± 9.3	0.163
Weight (kg)	76.1 ± 8.7	71.0 ± 6.3	75.4 ± 9.2	0.182
MRS (km/h)	33.1 ± 1.3	32.7 ± 1.6	31.5 ± 1.9	0.041
Match time exposure (min)	1321.5 ± 565.2	957.6 ± 740.0	1109.9 ± 501.9	0.298

## Data Availability

The original contributions presented in the study are included in the article, further inquiries can be directed to the corresponding author/s.

## Abbreviations

The following abbreviations are used in this manuscript:

MRS	Maximal Running Speed
GPS	Global Position System
ACTN-3	Alpha-actinin-3
RFLP	Restriction Fragment Length Polymorphism
PCR	Polymerase chain reaction
